# Tall stature in children: differential diagnosis and management

**DOI:** 10.1186/1687-9856-2013-S1-P53

**Published:** 2013-10-03

**Authors:** Sanjay Kumar

**Affiliations:** 1 Department of pediatrics, Sri Krishna Medical College & Hospital, Muzaffarpur, Bihar, India

## 

Tall stature is defined as height above 97th percentile for age and sex or more than 2SD above the mean for a defined population. It means 3 out of every 100 children in a community are tall and should be evaluated.

Familial tall stature also known as constitutional tall stature is the most common cause of tall stature. The height is consistently above 97th percentile percentile and mid paretal height too is above 90th or 97th percentile. Usually occurs in a female child and the mother often remembers her unusual tall stature during her childhood. The bone as is marginally to moderately advanced so that the final height prediction is not very heigh. Physical examination is normal and lab tests, if obtained, are negative.

The second most common cause of tall stature is nutritional. The height as well as the weight are at higher percentile. Again the bone age is maginally to moderately advanced so that final predicted adult height is not too much.

Hormonal causes of tall stature include hyperthyroidism, precocious puberty and growth hormone excess. Hyperthyroidism is more common in girls and is almost always caused by Grave's disease.The bone age is moderately advanced so that the final adult height is usually compromised.

In cases of precocious puberty, although due to anabolic effects of sex steroids, the child is tall at the onset, the ultimate adult height is compromised due to premature epiphyseal fusion caused by oestrogen. Again, although, delayed puberty may be associated with short stature in childhood, as with constitutional delay, failure to eventually enter puberty and complete sexual maturation may result in sustained growth in adult life, with ultimate tall stature [1].

GH hormone excess causes gigantism in childhhod and acromegaly in adults. Gigantism is characterized by tall stature, broad hands and feet, prognathism, broad root of nose, excessive sweating, hypertension and glucose intolerance. Almost each and every part of the body is affected and large for age. GH levels are consistently high and can exceed 100 ng/ml [2]. Serum IGF-1 and IGFBP-3 are raised and serve as a sensitive screening tool for GH excesses. But the gold standard for making the diagnosis of GH excess is the failure to suppress serum GH levels below 5 ng /dl after 1.75 gm/ kbw glucose challange. This test measures the ability of IGF-1 to suppress GH secretion, because the glucose load results in insulin secretion, leading to suppression of IGFBP-1 which results in an acute increase in serum free IGF-1 level.

There are some important chromosomal and syndromic causes of tall stature in children. Klinefelter’s syndrom (XXY) is the commonest chromosomal disorder affecting males (in 500-1000 boys). It should be suspected in boys with delayed puberty with tall stature, small and firm testes, eunochoid body proportions (decreased U/L segment) and gynaecomastia. And if these features are accompanied by delayed bone age it is almost diagnostic of Klinefelter syndrom. Serum levels of LH and FSH are raised and serum testosterone is markedly diminished.

Homocystinuria is an autosomal recessive inborn error of aminoacid metabolism due to deficiency of cystathionine synthase. The gene is located on chromosom 21. It is characterized by tall stature, arachnodactyly, mental retardation and various occular manifestations. There is characteristic inferior subluxation of lens. Lab investigation includes positive cyanide nitroprucide test in urine as well as presence of homocystein in the urine. This condition is to be differentiated from Marfan syndrom which is characterized by defective fibrillin gene on chromosom 15,resulting in abnormal synthesis of fibrillin, a glycoprotein that is the major constituent of microfibrills that provide scaffolding network of elastic fibres and have an anchoring function in non- elastic tissues such as aortic adventitia and suspensory ligaments of lens. Unlike homocystinuria there is no mental retadation and the subluxation of lens is upwards.

Sotos syndrom or cerebral gigantis is a non- endocrine condition of overgrowth which is characterized by large weight as well as height. The overgrowth continues even after birth so that by first birth day affected inants are greater than 97th percentile for height. Rapid growth continues till 4-5 years of age and then returns to the normal rate. The bone is advanced so the final adult height is in the upper normal range. There is no distinctive lab or radiological markers for the disease. Serum GH and IGF-1 levels are within normal limits.

Beckwith-weidman syndrome is a foetal overgrowth syndrome that is characterized by macrocephaly, macroglossia, omphalocoele, hepatosplenomegaly and hypoglycemia secondary to pancreatic B cell hyperplasia. In some cases, duplication of paternal IGF-2 gene with overexpression of IGF-2 has been seen [3]. Adult height is usually within normal range due to concomitant increase in bone age.

Base line investigations for tall stature:

• Karyotype

• T4,TSH

• IGF-1

• Bone age assessment and prediction of final height.

Special investigations include:

• Serum LH, FSH and testosterone levels

• Glucose suppression test for GH

• Vsual field examination

• MRI of pituitary

• Serum cortisol

• Serum prolactin

## Management of tall stature

Constitutional tall stature requires only reassurance after bone age assesment and prediction of final height. Obstinate cases require sex steroids to halt the progression of growth. In girls ethinylestradiol orally combined with cyclic progesterone has shown to reduce the final height upto 7 c.m.In boys testosterone 250-1000 mg monthly has shown simillar results.For best results these drugs should be started early i.e before 10 years in girls and 12.5 years in boys [4].

Nutritional tall stature is managed by life-style changes and avoidance of bad dietary practices.

Thyrotoxicosis is maned by use of antithyroid drugs. Methimazole and carbimazole are two commonly used antithyroid drugs.

Octreotides are somatostatin analogoues which can be used at a dose of 37.5-50 mg once or twice daily subcutaneously to reduce growth hormone hypersecretion and it has shown to reduce the final height upto 5cm [5].
